# Supplementary material for: MAP4K4/JNK Signaling Pathway Stimulates Proliferation and Suppresses Apoptosis of Human Spermatogonial Stem Cells and Lower Level of MAP4K4 Is Associated with Male Infertility
Source: Cells. 2022 Nov 28;11(23):3807. doi: 10.3390/cells11233807 (PMC9739186; doi:10.3390/cells11233807)
Supplement: Supplementary file 1 [file cells-11-03807-s001.zip › cells-1990359-supplementary.docx]

## Supplemental 4 Tables:

**Table S1. The sequences of MAP4K4 siRNA oligonucleotides**

| **siRNAs** | **Forward (5'-3')** | **Reverse (5'-3')** |
| --- | --- | --- |
| MAP4K4-siRNA1 | GGAAGGUCUAUCCUCUUAUTT | AUAAGAGGAUAGACCUUCCTT |
| MAP4K4-siRNA2 | GGCGGAGAAAUACGUUCAUTT | AUGAACGUAUUUCUCCGCCTT |
| Control siRNA | UUCUCCGAACGUGUCACGUTT | ACGUGACACGUUCGGAGAATT |

**Table S2. Primers used for RT-PCR and real time RT-PCR**

| **Genes** | **Forward primer (5'-3')** | **Reverse primer (5'-3')** | **Product sizes** |
| --- | --- | --- | --- |
| *PLZF* | GGACAAGGTTGAGGAAAGAGG | CAACACGGAGTAGATGCCCAG | 205 |
| *GPR125* | GCGTCATTACGGTCTTTGGAA | ACGGCAATTCAAGCGGAGG | 199 |
| *UCHL1* | AGCTGAAGGGACAAGAAGTTAG | TTGTCATCTACCCGACATTGG | 265 |
| *THY1* | CAGAAGGTGACCAGCCTAAC | TTGCTAGTGAAGGCGGATAAG | 233 |
| *RET* | CTCGTTCATCGGGACTTG | ACCCTGGCTCCTCTTCAC | 126 |
| *MAGEA4* | CTTACCCACTACCATCAGCTTC | TGATGACTCTCTCCAGCATTTC | 212 |
| *ACTB* | CCTGGCACCCAGCACAAT | GGGCCGGACTCGTCATAC | 144 |
| *SV40* | GAACAGCCCAGCCACTATAA | ACTCCAGCCATCCATTCTTC | 248 |
| *GFRA1*  *SPARC*  *ADAM19*  *GNG2*  *GPX7*  *COL1A1* | ACCAAGTACCGCACGCTAAG  CAATGACAACAAGACCTTCGAC  GGCTCTTCAGTTTACACAACAG  CAGGCAAAGTGTTTCTGAAAGA  CGCACCTACAGTGTCTCATTC  AAAGATGGACTCAACGGTCTC | TATGGGGAATCCTCCAGCAGA  GAATTCGGTCAGCTCAGAGTC  AAAGCTCCACATACTTCATGGA  TTCACAGTAGGCCATCAAATCT  CAGGTACTTGAAGGCAGGATG  CATCGTGAGCCTTCTCTTGAG | 233  151  86  164  81  181 |

**Table S3. The detailed information of antibodies used for immunocytochemistry and immunohistochemistry**

| **Antibodies** | **Dilution** | **Company** | **Catalog numbers** |
| --- | --- | --- | --- |
| UCHL1 | 1:50 | CST | 13179 |
| UCHL1 | 1:50 | Bio-rad | MCA4750GA |
| MAP4K4 | 1:200 | Bethyl | A301-502A |
| GFRA1 | 1:50 | RD | MAB7141 |
| GPR125 | 1:50 | Abcam | ab51705 |

**Table S4. The detailed information of the antibodies for Western blots**

| **Antibodies** | **Dilution** | **Companies** | **Catalog numbers** |
| --- | --- | --- | --- |
| ACTB | 1:1000 | CST | 8457S |
| MAP4K4 | 1:2000 | Bethyl | A301-502A |
| MAP4K4 | 1:500 | Proteintech | 55247-1-AP |
| GAPDH | 1:5000 | Proteintech | 10494-1-AP |
| PCNA | 1:1000 | CST | 13110S |
| p-JNK | 1:1000 | Abcam | ab124956 |
| JNK  Phosphor-MAP4K4 (Ser801) | 1:3000  1:500 | Proteintech  Bioss | 66210-1-Ig  Bs-5493R |

**Supplemental 4 Figures and Figure Legends**

**Supplemental Figure S1**

**
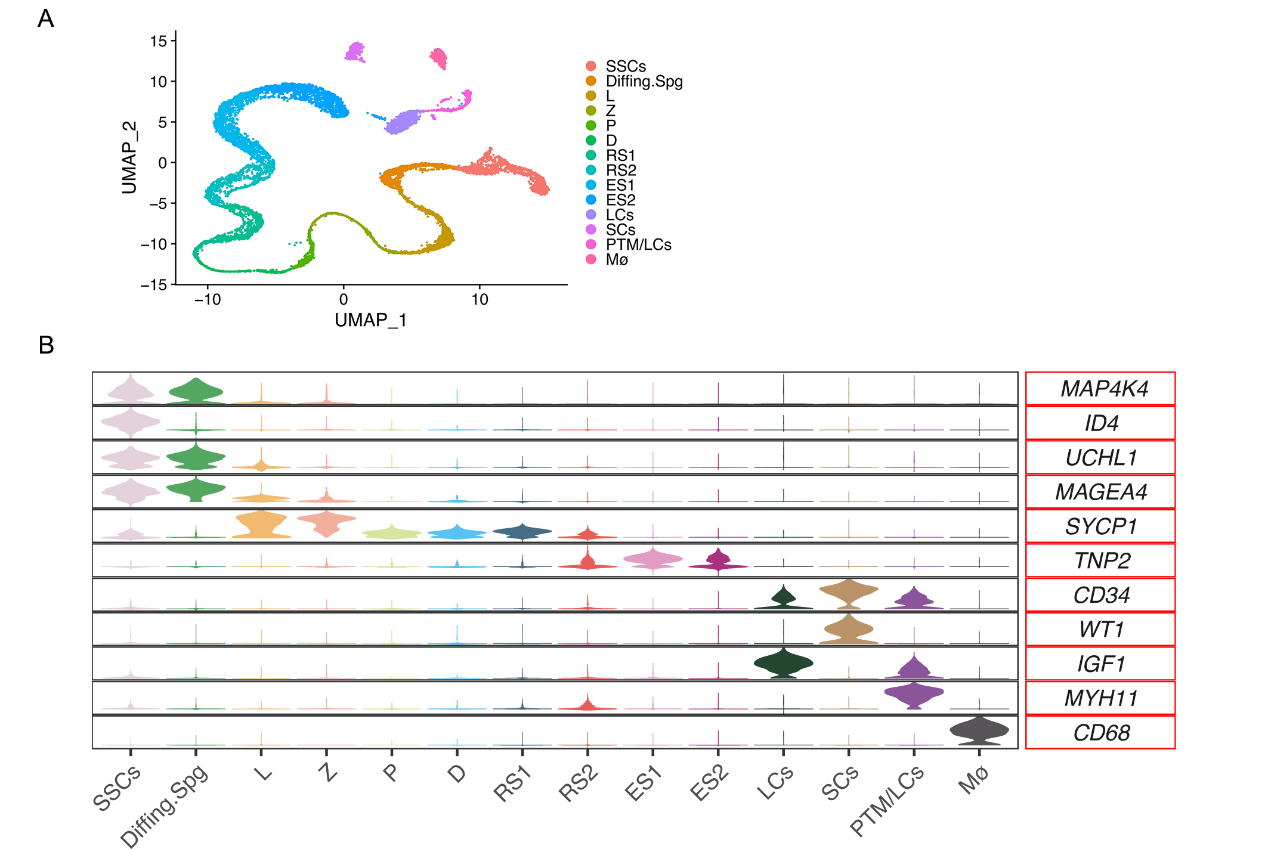
**

**Figure S1. UMAP and clustering analyses of testis single cell transcriptomic and the expression pattern of *MAP4K4* gene in all testicular cells.** (A) Testis single cell transcriptomic data from GSE109037 and GSE120508. (B) Violin plot showed the expression pattern of MAP4K4 gene in all testicular cells.

**Supplemental Figure S2**

**
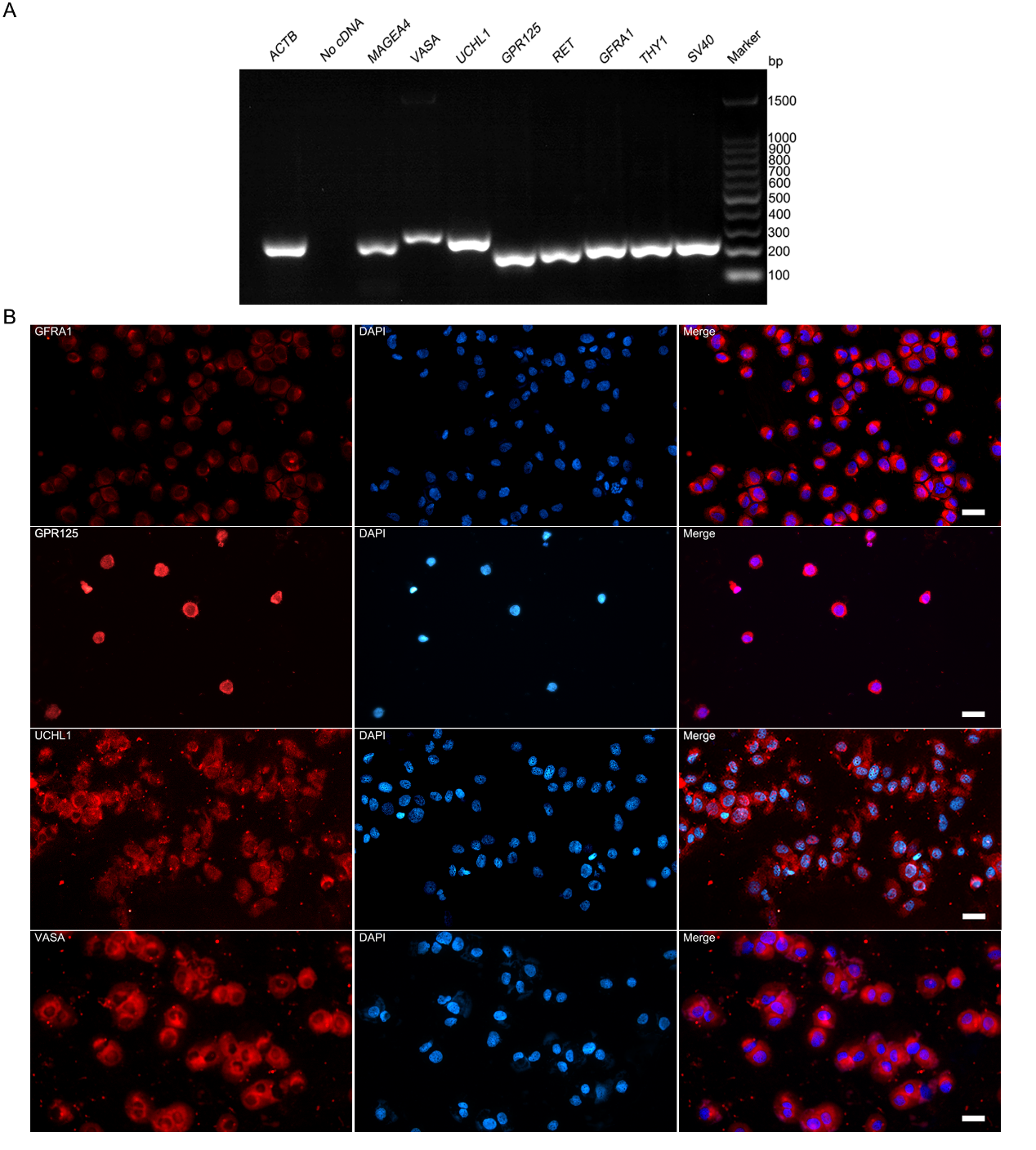
**

**Figure S2. Identification of the Human SSC line.** (A) RT-PCR revealed gene expression of *MAGEA4*, *VASA*, *UCHL1*, *GPR125*, *RET*, *GFRA1*, *THY1*, and *SV40* in human SSC line. No cDNA was used as a negative control and ACTB served as the loading control of total RNA. and. (B) Immunocytochemistry demonstrated the presence of GFRA1, GPR125, UCHL1 and VASA in human SSC line. Scale bars in B: 50 μm

**Supplemental Figure S3**


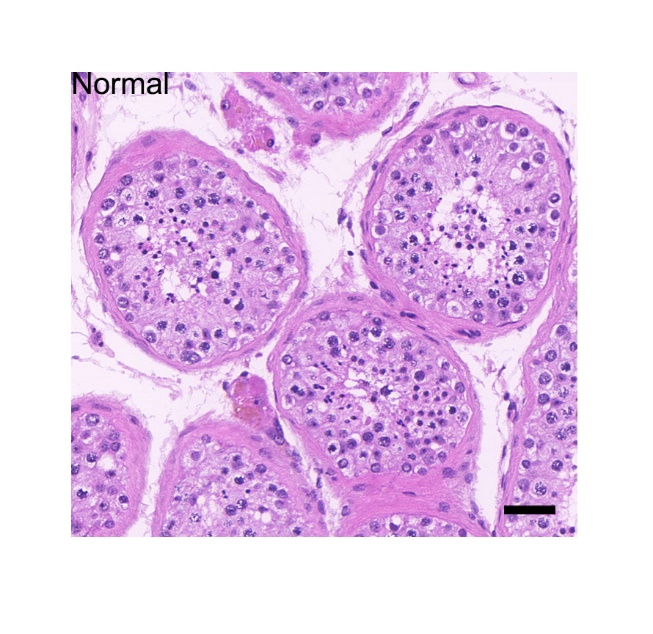


**Figure S3. H&E staining for testis with OA patients with normal spermatogenesis.** Testis sample from OA patients with normal spermatogenesis, and various germ cells were observed in seminiferous tubules. Scale bar: 50μm.

**Supplemental Figure S4**

**
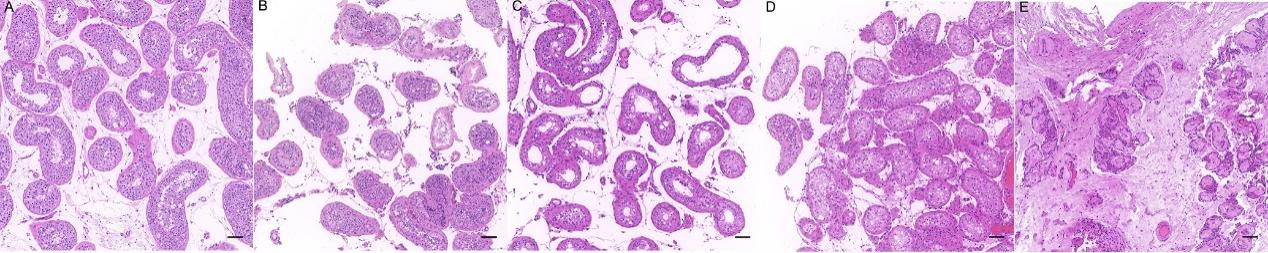
**

**Figure S4. H&E staining for testes of OA and NOA patients with germ cell maturation arrest.** (A, B) Testis samples from OA patients with normal spermatogenesis, and various types of male germ cells were observed in seminiferous tubules. (C) Testis samples from NOA patients with spermatocyte arrest, and there were a number of spermatocytes and no spermatid. (D, E) Testis samples from NOA patients with Sertoli cell only syndrome. All seminiferous tubules were lined by only Sertoli cells without any germ cells. Scale bars in A-E: 100 μm.
